# Association of *gyrA* and *rrs* gene mutations detected by MTBDR*sl* V1 on *Mycobacterium tuberculosis* strains of diverse genetic background from India

**DOI:** 10.1038/s41598-018-27299-z

**Published:** 2018-06-18

**Authors:** Syed Beenish Rufai, Jitendra Singh, Parveen Kumar, Purva Mathur, Sarman Singh

**Affiliations:** Division of Clinical Microbiology and Molecular Medicine, Department of Laboratory Medicine, All India Institute of Medical Sciences, New Delhi, India

## Abstract

There is limited data on the use of Genotype MTBDR*sl*Version 1 (MTBDR*sl* V1) as an initial rapid screening test to rule out XDR-TB and most importantly its performance in various genotypes of *Mycobacterium tuberculosis* is scarcely studied. A total of 359 MDR-TB isolates were tested for gene mutations representing second line drug resistance, using the MTBDR*sl*_V.1 and the results were compared with phenotypic method (Bactec MGIT-960 system) for second-line drug (SLD) susceptibility testing. Genetic lineages of all these isolates were also determined using spoligotyping and SITVIT2 WEB database. The MTBDR*sl* V1 detected mutations in the *gyrA*, *rrs*, and *emb* genes in 108 (30%), 2 (0.5%) and 129 (35.9%) isolates, respectively. Remaining 120 (33.4%) had no second line drug (SLD) resistance. In 17 (4.7%) isolates mutations were detected in both *gyrA* and *rrs* genes. Its concordance with MGIT-960 culture drug susceptibility testing (DST) was 97% and 94.1%, 93.5%, 60.5% and 50% for the detection of XDR-TB, pre-XDR, Ethambutol, and Aminoglycosides/Cyclopeptides resistance. The Beijing lineage was predominant (46%) between both the pre-XDR/XDR-TB isolates. We conclude that MTBDR*sl* is useful for rapid detection of SLD resistance. Also in pre-XDR and XDR-TB isolates the frequency of relevant genetic mutations was significantly higher in the Beijing strains.

## Introduction

The recent estimates of World Health Organization (WHO) show that more than 10.4 million people had incidental tuberculosis (TB) and 1.7 million died of this disease in 2016 alone. The 2016 data also showed that India is the most vulnerable country to TB with 2.8 million new cases (26.9% of global cases) in the year 2016^1^. The scenario was not much different in 2017. Approximately 600,000 Rifampicin resistant (RIF^R^) cases were reported, of whom 490,000 had multi-drug resistant TB (MDR-TB). The drug resistance TB has become a major challenge to the success of TB control programmes around the globe^2^. Not only diagnosis, even the treatment of MDR-TB is difficult, requiring much longer duration of treatment with very costly and comparatively more toxic second-line drugs (SLDs) such as fluoroquinolones (FQ) and aminoglycosides/cyclic peptides (AG/CP)^3^. Moreover, emergence of resistance even to the SLDs is being reported from all parts of the world. This form of TB is known as extensively drug-resistant TB (XDR-TB), which is a more devastating condition with very poor treatment success^4^. In 2016, an estimated 6.2% patients with MDR-TB were found to have XDR-TB. India reported first case of XDR-TB^5^ in 2007 and by 2015 more than 117 countries were already struggling with this condition^1,5^.

Therefore, it is essential that all suspected cases of MDR-TB must be investigated for susceptibility to second drugs also, in a timely manner to control the spread of spread of XDR-TB. This has become essential in order to optimize the treatment regimen at the earliest. However, the conventional methods of drug susceptibility testing (DST) for SLDs are more complex due to non-standardized methods and protocols, leading to poor reproducibility and reliability^6^. Also, the conventional culture-based methods are labour intensive and require longer turn around time (TAT) with undesirable treatment outcome and wider window to disease transmission^7,8^.

In 2013, a Line probe assay (LPA), also known as Genotype MTBDR*sl* (MTBDR*sl*) was developed by Hain’s Life Science GmbH, Germany and was approved by the WHO also for clinical use. This molecular test rapidly detects genotypic resistance to FQ, AG/CP and Ethambutol (EMB) within 48–72 h and makes it possible to diagnose pre-XDR-TB and XDR-TB at the earliest^9,10^. Therefore, the use of MTBDR*sl* has been recommended as a rapid and initial diagnostic test to rule out FQ and AG/CP resistance in all MDR-TB patients in order to initiate effective treatment at the earliest^11^. Recently, WHO recommended the use of MTBDR*sl* test in place of conventional phenotypic culture-based DST methods especially in high TB burden countries^12^. India, a high TB burden country is keen to implement these guidelines but usefulness of MTBDR*sl* on MDR TB isolates has not yet been evaluated from India.

The genotypic characterization of MTB isolates becomes essential to understand the clonal expansion of the lineages, their transmission dynamics and association with drug resistance^13^. There are several studies which have shown association of different lineages of MTB with variable pathogenicity and vulnerability to drug resistance^14–17^. For genotyping of the MTB, spoligotyping is a widely used technique which detects the presence or absence of 43 spacer sequences in clustered regularly interspersed short palindromic repeat (CRISPR) region of MTB^14^. Though several studies are published on association of various lineages of MTB with first-line drug resistance, there is not enough literature on the association of MTB lineages with resistance to second-line drugs^14^.

Thus, the main aim of the present study was to evaluate the usefulness of MTBDR*sl* assay in comparison with phenotypic line drug resistance testing using the Bactec MGIT-960 system in a programmatic mode. We also aimed to find if there was any association between the second line drug resistance pattern and genetic lineages of MTB isolates.

## Materials and Methods

### Setting

This retrospective study was conducted in the TB research laboratory, which is a certified routine diagnostic laboratory in the Division of Clinical Microbiology & Molecular Medicine, All India Institute of Medical Sciences, New Delhi, India. All the routine mycobacterial isolates are stored and maintained in laboratory repository after characterization. From this repository, 359 MDR-TB isolates that were stored during 2011–2015 were used in the present study. Patient’s clinical details were retrieved as published earlier^14,18^ (Table 1). These isolations were made as a part of previous study, which was approved by Institutional ethics committee of the All India Institute of Medical Sciences, New Delhi (reference number IESC/T-39/04.01.2013). All *in-vitro* methods were performed in accordance with the standard guidelines and following the manufacturer’s instructions. The mycobacterial culture and DST were performed using the MGIT-960 system and identification of *Mycobacterium tuberculosis* (MTB) and Non-tuberculous Mycobacteria (NTM) was done by well established *in-house* multiplex polymerase chain reaction (PCR)^19^.

**Table 1:** Showing association of lineages with place of origin, smear positivity, site of isolation and drug resistance.

Main Residence of patient	Smear microscopy		Site of sample		Drug resistance Pattern			Genotypes									
	Smear + ve	Smear −ve	PTB	EPTB	Sensitive	Pre-XDR	XDR	Beijing	CAS	EAI	Manu	T	X	S	H	Ural	Unique
Assam (44)	44	0	44	0	20	21	3	27	9	2	1	1	1	0	0	0	3
Bihar (35)	12	23	15	20	23	10	2	10	13	0	1	3	0	0	2	1	5
Delhi (132)	61	71	74	58	98	31	3	25	67	2	5	12	6	0	8	0	7
Haryana (5)	1	4	2	3	4	1	0	2	1	0	0	0	0	0	1	0	1
J&K (1)	0	1	0	1	0	1	0	0	0	0	0	0	0	0	1	0	0
Meghalaya (20)	20	0	20	0	14	5	1	14	5	0	0	1	0	0	0	0	0
Mizoram (2)	0	2	2	0	2	0	0	0	0	0	0	0	0	2	0	0	0
Madhya Pradesh (1)	0	1	0	1	0	1	0	0	1	0	0	0	0	0	0	0	0
Punjab (70)	70	0	70	0	42	23	5	18	34	4	0	6	0	0	0	0	8
Rajasthan (1)	0	1	0	1	0	1	0	0	0	0	0	0	0	0	0	0	1
Sikkim (10)	8	2	8	2	7	3	0	6	2	1	0	0	0	0	1	0	0
Tripura (13)	13	0	13	0	6	5	2	10	2	1	0	0	0	0	0	0	0
Uttar Pradesh (24)	11	13	11	13	15	8	1	6	8	2	0	3	1	0	0	1	3
Uttarakhand (1)	0	1	0	1	0	1	0	1	0	0	0	0	0	0	0	0	0
**Total (359)**	**240**	**119**	**259**	**100**	**231**	**111**	**17**	**119**	**142**	**12**	**7**	**26**	**8**	**2**	**13**	**2**	**28**

PTB- Pulmonary Tuberculosis.

EPTB- Extra-pulmonary Tuberculosis.

### Demographic details and characteristics of MDR-TB patients

Out of the 359 patients from whom these isolations were made, 210 (58.5%) were males and 149 (41.5%) females with mean age of 31.4 ± 14.3 years and 27.1 ± 15.8 years, respectively. Majority of cases were adults [331 (92.2%)] and only a few [28(7.8%)] were from paediatric age group. Majority of isolates [259 (72.1%)] were from pulmonary samples and 100 (27.8%) isolates were from extra-pulmonary samples (Supplementary Fig. 1). A total of 240 (66.8%) isolates were from smear positive while 119 (33.2%) isolates were from smear negative samples (Table 1).

### Subculture on Lowenstein Jensen (L-J) medium for pure growth

From each Bactec MGIT-960 tube, 200 μL of culture suspension was sub-cultured on L-J medium slants and incubated at 37°C to obtain pure growth (isolated colonies) of MTB. After 21–28 days of incubation, single colony with the help of sterile inoculating loop was picked from L-J medium and inoculated in the MGIT (Mycobacteria growth indicator tube). The tube was incubated in the Bactec MGIT-960 system until flagged positive and this growth was used for second-line MGIT DST and DNA extraction for spoligotyping and MTBDR*sl*^20^.

### Preparation of drug stock and working solutions

All SLDs were purchased from Sigma-Aldrich Corporation (St. Louis MO, USA) in the form of powder. The stock solutions of amikacin (AMK), kanamycin (KAN), and capreomycin (CAP) were prepared in sterile deionized water while ofloxacin (OFX) solution was prepared in 0.1N-NaOH. Stock solutions were sterilized through 0.22-μM-pore-size Milex-GS filter units (Millipore Bedford MA, USA) and the aliquots stored at −80 °C for further use.

### Second Line Drug susceptibility (DST) testing using Bactec MGIT-960 system

Aliquots of OFX, AMK, KAN and CAP stock solutions were diluted to critical concentrations as recommended to perform the second line DST by Bactec MGIT-960 system^7^. DST was performed on Day 1 and Day 2 by single dilution [0.5 mL of 1:100 dilution inoculum for growth control (GC) and 0.5 mL of inoculums directly in four respective drug containing tubes] while from the growth of Day 3 to Day 5 by double dilution [inoculated 0.5 mL of 1:4 dilution inoculums directly into four drug containing tubes] and in GC tubes using the 1:100 dilution of the inocula from the day MGIT flashed positive. GC tubes and four drug panel tubes were set in the antimicrobial susceptibility testing (AST) carrier rack and loaded in the Bactec MGIT-960 system and continuously monitored by BD Epi-center^21^. As AST carrier rack for SLDs, the panel is not available commercially for the MGIT-960 system, it was registered as one of the SIRE (Streptomycin, Isoniazid, Rifampicin, Ethambutol) panel in order to get a printable report and drug susceptibility testing results^22^.

### Genotype MTBDR*sl* V1

The MTBDR*sl* test is based on the DNA strip technology having three steps: DNA extraction, multiplex PCR amplification, and reverse hybridization. All steps were performed as per manufacturer’s instructions^10,23^. The DNA obtained from the standard MTB- *H37RV* strain (as positive control) and one negative control was also tested in each batch in order to check the cross-contamination during hybridization assay and other quality parameters. The test was considered as valid; only when the hybridization bands were obtained on MTB complex control (TUB), conjugate controls (CC) and the amplification controls (AC) along with the targeted gene loci controls. For convenience MTBDR*sl* V1 will be referred as MTBDR*sl* only hereafter.

### Genotyping of MDR-TB isolates by spoligotyping

#### DNA extraction for spoligotyping

DNA extraction from MTB cultures grown on L-J medium was performed using chloroform iso-amyl alcohol (CI) method as mentioned previously^24^.

### Spoligotyping

Spoligotyping was performed using the commercially available kit (Ocimum Biosolutions, Hyderabad, India) by amplification, hybridization and finally detection of hybridizing DNA^25^. In brief, the PCR amplified products were hybridized on a membrane and images were detected with electro-chemi-luminescence (ECL) detection kit (GE Healthcare, Life Sciences, UK) on X-ray films (Kodak, Rochester, NY)^14^. The MTB *H37Rv* and *M*. *bovis*-BCG strains were included as a quality control in every batch of test. The hybridization patterns obtained in the binary format were transformed to an octal code for assessment with the spoligotype patterns using SITVIT2 database, which is an updated version of SITVIT_WEB database^26^. A shared type was defined as a spoligotype pattern common to at least two isolates, and clades were assigned according to the signatures as published earlier^14^.

### Statistical analysis

Results of MTBDR*sl* were analysed and compared with second-line Bactec MGIT-960 DST, which was considered as the gold standard. Data was statistically analysed to calculate the agreement between MTBDR*sl* and SL-MGIT DST using OpenEpi 3.01. Moreover, Fisher’s exact test was performed using STATA 11.1 software to observe significance of the association amongst the second line drug resistant mutation patterns among the different genotypes.

## Results

### Second-line DST by Bactec MGIT-960 system

Using the phenotypic MGIT-960 system, of the 359 MDR-TB isolates subjected to second-line DST, 231 (64.4%) were found to be susceptible to all second line anti-TB drugs, 127 had resistance to FQ. Of the 127 isolates, 110 (30.6%) were mono-resistant to FQ (pre-XDR) and 10 (2.7%) isolates were resistant to all OFX-KAN-AMK-CAP, 6 (1.7%) were resistant to OFX-KAN-AMK and 1 (0.3%) isolate was resistant to OFX-KAN only. Thus, phenotypically these 17 (4.7%) isolates were labelled as XDR-TB isolates. Only one (0.3%) isolate was resistant to AG/CP (Table 2).

**Table 2:** Concordance between Genotype MTBDR*sl* and Bactec MGIT-960 based second-line drug susceptibility testing.

**Genotype MTBDR** ***sl***	**SLD by Bactec MGIT 960**					
	Sensitive **(%)**	FQ mono-resistant **(%)**	AG/CP mono-resistant **(%)**	XDR **(%)**	Concordance **(%)**	***k*** **coeff**
Sensitive (232) (64.6%)	225 (97)	7(3.0)	—	—	97	0.92 Perfect
FQ- resistant (108) **(30%)**	6(5.5)	101 (93.5)	—	1(0.9)	93.5	0.89 Perfect
AG/CP- resistant (2) **(0.5%)**	—	1(50)	1 (50)	—	50	0.66 Substantial
XDR (17) (4.7%)	—	1(5.9)	—	16(94.1)	94.1	0.93 Perfect
Total (359)	231 (64.4)	110 (30.6)	1 (0.3)	17 (4.7)	—	

FQ- Fluoroquinolone, AG- Aminoglycosides, CP-Cyclopeptide.

*k* coeff.- Cohens’s kappa as a measure of agreement between two values.

### EMB resistance

All 359 isolates previously tested for SIRE DST, 143 (39.8%) isolates were detected as resistant and 216 (60.2%) isolates were detected sensitive to EMB (Supplementary Table 1).

### Genotype MTBDR*sl*V1

Out of 359 MDR-TB isolates, in 232 (64.6%) isolates no mutation was detected in *gyrA* and *rrs* genes, which means that these were sensitive to FQ and AG/CP drugs. However, 125 (34.8%) isolates showed mutations in *gyrA* region, of which 108 showed FQ mono-resistant (pre-XDR) but 17 also showed additional mutations in *rrs* genes (XDR-TB) (Table 2). Among the single codon mutations observed in *gyrA* region, the most prevalent mutation was ΔWT3-D94G (51; 40.8%) followed by ΔWT2-A90V (31; 24.8%). However, among the double codon mutations, the most prevalent mutation was A90V-D94G (7; 5.6%) followed by ΔWT3-D94G-D94H (2; 1.6%). Only one strain harboured triple codon mutation ΔWT3-D94N/Y-D94H-D94G in *gyrA* region (Table 3).

**Table 3:** Association of genotypes with mutational pattern in *gyrA* (108 FQ mono-resistant and 17 XDR-TB) and *rrs* gene regions (2 AG/CP mono-resistant and 17 XDR-TB isolates) detected by Genotype MTBDR*sl* assay on 125 culture isolates.

Codon mutation *(gyrA)*	Total no. of isolates (%)	Genotype (%)								
		Beijing	CAS	EAI	H	Manu	T	Ural	Unique	X
ΔWT3-D94G	51 (40.8)	23 (45.1)	14 (27.4)	—	1 (1.9)	1 (1.9)	3 (5.8)	—	4 (7.8)	5 (9.8)
ΔWT3-D94A	6 (4.8)	4 (66.7)	1 (16.7)	—	—	—	—	—	1 (16.6)	—
ΔWT3-D94N/Y	6 (4.8)	6 (100)	—	—	—	—	—	—	—	—
ΔWT3-D94H	5 (4)	3 (60)	2 (40)	—	—	—	—	—	—	—
ΔWT3	3 (2.4)	2 (66.7)	1 (33.3)	—	—	—	—	—	—	—
ΔWT2-A90V	31 (24.8)	10 (32.2)	8 (25.8)	3 (9.7)	—	3 (9.7)	2(6.4)	2 (6.4)	2 (6.4)	1(3.2)
ΔWT2-S91P	1 (0.8)	1 (100)	—	—	—	—	—	—	—	—
+WT-A90V	3 (2.4)	2 (66.7)	—	—	—	—	1(33.3)	—	—	—
+WT-D94G	2 (1.6)	—	2 (100)	—	—	—	—	—	—	—
+WT-D94H	2 (1.6)	2 (100)	—	—	—	—	—	—	—	—
+WT-D94A	1 (0.8)	—	—	—	—	—	1 (100)	—	—	—
ΔWT3-D94G- D94H	2 (1.6)	—	—	—	—	—	—	—	2 (100)	—
A90V, D94A	2 (1.6)	1 (50)	—	1 (50)	—	—	—	—	—	—
+WT-A90V- D94G	7 (5.6)	2 (28.6)	3 (42.8)	—	—	—	—	—	2 (28.5)	—
+WT-A90V-D94A	1 (0.8)	—	1 (100)	—	—	—	—	—	—	—
ΔWT3-D94A- D94G	1 (0.8)	—	—	—	—	—	1 (100)	—	—	—
ΔWT3-D94N/Y- D94G-D94H	1 (0.8)	—	1 (100)	—	—	—	—	—	—	—
Total:	125 (34.8)	56 (44.8)	33 (26.4)	4 (3.2)	1 (0.8)	4 (3.2)	8(6.4)	2 (1.6)	11 (8.8)	6 (4.8)
**Codon Mutation (** ***rrs*** **)**										
ΔWT1-A1401G	11 (57.9)	6 (54.5)	2 (18.2)	1 (9.1)	—	—	1 (9.1)	—	1 (9.1)	—
ΔWT1-G1484T	1 (5.3)	—	—	—	—	—	—	—	—	1 (100)
ΔWT1-C1402T	1 (5.3)	1 (100)								
+WT-A1401G	6 (31.6)	3 (50)	2 (33.3)	—	—	—	—	1 (16.7)	—	—
**Total:**	**19 (5.2)**	**10 (52.6)**	**4 (21.1)**	**1 (5.3)**	—	—	**1 (5.3)**	**1 (5.3)**	**1(5.3)**	**1 (5.3)**

ΔWT- Deletion of wild type band. +WT- Presence of wild type band.

Of the 19 (15.2%) isolates that showed resistance mutation pattern in the *rrs* region, 17 (89.4%) were XDR-TB and 2 (10.6%) were AG/CP mono-resistant. In these isolates, most prevalent mutation was ΔWT1-A1401G (11; 57.9%) and A1401G (31.6%). The detailed mutational patterns in *gyrA* and *rrs* genes are shown in Table 3.

### Detection of EMB resistance by Genotype MTBDR*sl* V1 in *emb* gene

Overall MTBDR*sl* detected 129 (35.9%) isolates having resistance to EMB. Of these 58 (44.6%) isolates were mono-resistant to FQ, 1 (0.7%) isolate was mono-resistant to AG/CP only and 9 (6.9%) isolates were XDR-TB isolates. The most prevalent mutations in the *emb* gene were ΔWT1-Mut1b in 95 (73.6%) isolates, ΔWT1-Mut1a in 20 (15.5%), ΔWT in 10 (7.7%), and ΔWT1-Mut1a-Mut1b in 4 (3.1%) isolates.

### Comparison of second line DST using MGIT-960 versus Genotype MTBDR*sl* V1

The MTBDR*sl* showed 97% concordance with second-line MGIT-960 DST for detecting the sensitive isolates. However, mutations were detected in 101 (93.5%) isolates that were FQ resistant by MGIT-960 system. MTBDR*sl* also efficiently detected 17 (4.7%) isolates as XDR-TB giving a concordance rate of 94.1% with MGIT-960 system (Table 2). Of the 143 (39.8%) EMB resistant isolates identified by SIRE MGIT-960 DST, 86 (60.1%) were detected resistant and 57 (39.9%) as sensitive by the MTBDR*sl*. Thus, the overall concordance of MTBDR*sl* with MGIT-960 system was only 50% for detection of EMB resistance.

### Genotyping Results

The spoligotyping results showed that 142 (39.5%) isolates belonged to CAS lineage, 119 (33.2%) Beijing, 26 (7.2%) T, 13 (3.6%) EAI, 12 (3.3%) Haarlem, 8 (2.2%) X, 7 (1.9%) Manu and 2 (0.5%) isolates each of Ural and S lineages. Twenty-eight (7.7%) isolates showed Unique (U) patterns. Out of 359 MDR-TB isolates, 322 (89.7%) were grouped in 55 SITs (Shared international types). Nine (2.5%) strains were identified as “orphans”. The remaining 28 (7.7%) isolates could not be categorized in any SITs (Table 4). Among the 55 STs (share types), the two most common were ST1 (Beijing) [111 (30.9%)] and ST26 (CAS1_DELHI) [84 (23.4%)] followed by ST25 (CAS1_DELHI, 3.3%) and ST53 (T1, 2.5%). The remaining SITs represented less than 2.5% isolates. Out of 9 orphans, 2 (0.5%) each belonged to CAS, T1, and Manu lineage and 1 (0.3%) each isolate belonged to CAS1_DELHI, T1, and H4 families (Table 4).

**Table 4:** Spoligotyping pattern, octal codes, SIT and lineage of overall MDR-TB isolates use

SIT	SPOLIGOTYPE DESCRIPTION	OCTAL CODE	LINEAGE	RESISTANT TYPE			Total (%)
				MDR (N = 231)	Pre-XDR (N = 111)	XDR (N = 17)	
1	□□□□□□□□□□□□□□□□□□□□□□□□□□□□□□□□□□■■■■■■■■■	000000000003771	BEIJING	57 (24.7)	46 (41.4)	8 (47.1)	111 (30.9)
11	■□□■■■■■■■■■■■■■■■■■■■■■■■■■□□□□■□■■□□□■■■■	477777777413071	EAI3_IND	4 (1.7)	1 (0.9)	0	5 (1.4)
25	■■■□□□□■■■■■■■■■■■■■■■□□□□□□□□□□□□■■□□■■■■■	703777740003171	CAS1 DELHI	11 (4.7)	1 (0.9)	0	12 (3.3)
26	■■■□□□□■■■■■■■■■■■■■■■□□□□□□□□□□□□■■■■■■■■■	703777740003771	CAS1 DELHI	65 (28.1)	18 (16.2)	1 (5.8)	84 (23.4)
27^**^	■■■□□□□■■■■■■■■■■■■■■■□□■■■■■■■■■□□□□■■■■■■■	703777747770371	URAL	0	1 (0.9)	0	1 (0.3)
34	■■■■■■■■□□■■■■■■■■■■■■■■■■■■■■■■■□□□□■■■■■■	776377777760771	S	2 (0.9)	0	0	2 (0.6)
37	■■■■■■■■■■■■□■■■■■■■■■■■■■■■■■■■□□□□■■■■■■■	777737777760771	T3	1 (0.4)	0	0	1 (0.3)
48	■■■■■■■■■■■■■■■■■■■■■■■■■■■■□□□□■□■■■■■□■■■	777777777413731	EAI2_SOM	0	2 (1.8)	0	2 (0.6)
52	■■■■■■■■■■■■■■■■■■■■■■■■■■■■■■■■□□□□■■■□■■■	777777777760731	T2	1 (0.4)	0	0	1 (0.3)
53	■■■■■■■■■■■■■■■■■■■■■■■■■■■■■■■■□□□□■■■■■■■	777777777760771	T1	9 (3.9)	0	0	9 (2.5)
54	■■■■■■■■■■■■■■■■■■■■■■■■■■■■■■■■□□■■■■■■■■■	777777777763771	MANU2	2 (0.9)	2 (1.8)	0	4 (1.1)
67	■■■■■■■■■■■■□■■■■■□□□□■■■■■■■■□■□□□□■■■■■■■	777777037720771	H3	4 (1.7)	1 (0.9)	0	5 (1.4)
92	■■■□□□□□□□□□■■■■■□■■■■■■■■■■■■■■□□□□■■■■■■■	700076777760771	X3	1 (0.4)	0	0	1 (0.3)
100	■■■■■■■■■■■■■■■■■■■■■■■■■■■■■■■■■□■■■■■■■■■	777777777773771	MANU1	1 (0.4)	0	0	1 (0.3)
119	■■■■■■■■■■■■■■■■■□■■■■■■■■■■■■■■□□□□■■■■■■■	777776777760771	X1	0	4 (3.6)	1 (5.8)	5 (1.4)
125^**^	□□□□□□□□□□□□□□□□□□□□□□□□□■■■■■■■■□□□□■■■□■■■	000000007760731	T2	0	1 (0.9)	0	1 (0.3)
127	■□■■■■■■■■■■■■■■■■■■■■■■■■■■□□□■□□□□■■■■■■■	577777777420771	H4	2 (0.9)	0	0	2 (0.6)
137	■■■■■■■■■■■■■■■■■□■■■■■■■■■■■■■■□□□□■■□□□□■	777776777760601	X2	0	1 (0.9)	0	1 (0.3)
190^**^	□□□□□□□□□□□□□□□□□□□□□□□□□□□□□□□□□□■■■■■□■■■	000000000003731	BEIJING	0	2 (1.8)	0	2 (0.6)
236	■■■■■■■■■■■■■■■■■■■■■■■■■■■■□□□□■□■■■■■■■■■	777777777413771	EAI5	0	0	1 (5.8)	1 (0.3)
243	■■■■■■■■■■■■■■■■■■■■■■■■■■■■■■■■□□□□■■□□□□□	777777777760600	T1	2 (0.9)	2 (1.8)	0	4 (1.1)
250^*^	□□□□□□□□□□□□□□□□□□□□□□□□□□□□□□□□□□□□□■■■■■■	000000000000371	BEIJING	2 (0.9)	0	0	2 (0.6)
283	■■■■■■■■■■■■■■■■■■■■■□□□■□□□□□□■□□□□■■■■■■■	777777704020771	H1	3 (1.3)	1 (0.9)	0	4 (1.1)
288	■■■□□□□□□□■■■■■■■■■■■■□□□□□□□□□□□□■■■■■■■■■	700377740003771	CAS2	1 (0.4)	1 (0.9)	0	2 (0.6)
289	■■■□□□□■■■■■■■■■■■■■■■□□□□□□□□□□□□■■■□■■■■■	703777740003571	CAS1 DELHI	2 (0.9)	0	0	2 (0.6)
344	■■■□□□□□□□□□■■■■■■■■■■■■■■■■■■■■□□□□■■■■■■■	700077777760771	T1	1 (0.4)	0	0	1 (0.3)
357	■■■□□□□■■■■■■■■■■■■■■■□□□□□□□□□□□□□□■■■■■■■	703777740000771	CAS	3 (1.5)	0	0	3 (0.8)
358^**^	■■■□□■■■■■■■■■■■■■■■■■■■■■■■■■■■□□□□■■■■■■■	717777777760771	T1	0	1 (0.9)	0	1 (0.3)
428	■■■□□□□■■■■■■■■■■■■■■■□□□□□□□□□□□□■■□■■■■■■	703777740003371	CAS1 DELHI	2 (0.9)	0	0	2 (0.6)
429	■■■□□□□■■■■■■■■■■■■■■■□□□□□□□□□□□□■■■■■□■■■	703777740003731	CAS1 DELHI	0	1 (0.9)	1 (5.8)	2 (0.6)
462	■■■■■■■■■■■■■■■■■■■■■■■■■■■■■□■■■□□□□■■■■■■	777777777560771	T1	1 (0.4)	0	0	1 (0.3)
464^***^	■■■■■■■■■■■■■■■■■■■■■■■■■■■■■■■■□■■■■■■■■■■	777777777747771	URAL	0	0	1 (5.8)	1 (0.3)
486	■■■□□□□■■■■■■■■■■■■■■■□□□□□□□□□□□□□□□■■■■■■	703777740000371	CAS	2 (0.9)	1 (0.9)	0	3 (0.8)
591	■■■■■■■■■■■■■■■■■■■■■■□■■■■■□□□□■□■■■■■■■■■	777777757413771	EA6_BGD1	2 (0.9)	0	0	2 (0.6)
621	□□□□□□□□□□□□□□□□□□□□□□□□□□□□□□□□□□■□■■■■■■■	000000000002771	BEIJING	0	0	1 (5.8)	1 (0.3)
754	■□■□□□□■■■■■■■■■■■■■■■□□□□□□□□□□□□■■■■■■■■■	503777740003771	CAS1 DELHI	0	2 (1.8)	0	2 (0.6)
794	■■■□□□□■■■■■■□■■■■■■■■□□□□□□□□□□□□■■■■■■■■■	703757740003771	CAS1 DELHI	2 (0.9)	2 (1.8)	0	4 (1.1)
798^**^	■□□□■■■■■■■■■■■■■■■■■■■■■■■■■■■■□□□□■■■■■■■	437777777760771	T1	0	1 (0.9)	0	1 (0.3)
1091	■■■□□□□■■■■■■■■■■■■■■■□□□□□□□□□□□□■□■■■■■■■	703777740002771	CAS1 DELHI	1 (0.4)	1 (0.9)	0	2 (0.6)
1092	■■■□□□□■□■■■■■■■■■■■■■□□□□□□□□□□□□■■■■■■■■■	702777740003771	CAS1 DELHI	1 (0.4)	0	0	1 (0.3)
1120	■■■□□□□■■■■■■■□□□□□□□□□□□□□□□□□□□□□□□■■□■■■	703760000000331	CAS	2 (0.9)	0	1 (5.8)	3 (0.8)
1166^**^	■■■■■■■■■□■■■■■■■■■■■■■■■■■■■■■■□□□□■■■■■■■	777377777760771	T1	0	1 (0.9)	0	1 (0.3)
1168^*^	□□□□□□□□□□□□□□□□□□□□□□□□□□□□□□□□□□■■■■□□■■■	000000000003631	BEIJING	3 (1.3)	0	0	3 (0.8)
1327	■■■□□□□■■■■□□■■■■■■■■■□□□□□□□□□□□□■■■■■■■■■	703637740003771	CAS1 DELHI	1 (0.4)	2 (1.8)	0	3 (0.8)
1343	■■■□□□□■■■■■□■■■■■■■■■□□□□□□□□□□□□■■■■■■■■■	703737740003771	CAS1 DELHI	2 (0.9)	0	0	2 (0.6)
1394^*^	■■■■■■■■■■■■■■■■■□■■■■■■■■■■■■■■□□□□■■□■■■■	777776777760671	X1	1 (0.4)	0	0	1 (0.3)
1401^*^	■■■□□□□■■■■■■■■■■■■■■■□□□□□□□□□□□□■■■■□■■■■	703777740003671	CAS1 DELHI	3 (1.3)	0	0	3 (0.8)
1628^*^	□□□■■■■■■■■■□■■■■■■■■■■■■■■■□□□□■□■■■■■■■■■	077737777413771	EAI5	1 (0.4)	0	0	1 (0.3)
1789	■■■□□□□■■■■■■■■■■■■■■■□□□□□□□□□□□□□□□□■■■■■	703777740000171	CAS	2 (0.9)	0	0	2 (0.6)
1877	■■■□■■■■■□■■■■■■■■■■■■■■■■■■■■■■□□□□■■■■■■■	737377777760771	T1	0	1 (0.9)	1 (1.8)	2 (0.6)
1942	■■■□□□□■■□■■■■■■■■■■■■□□□□□□□□□□□□■■■■■■■■■	703377740003771	CAS1 DELHI	1 (0.4)	0	0	1 (0.3)
1970	■■■■■■■■■■■■■■■■■□■■■■□■■■■■□□□□■□■■■■■■■■■	777776757413771	EAI6_BGD	2 (0.9)	0	0	2 (0.6)
2147^**^	■■■□□□□■■■■■■■■■■■■■■■□□□□□□□□□□□□■■□□□□□■■	703777740003011	CAS1 DELHI	0	2 (1.8)	0	2 (0.6)
2364	■■■□□□□■■■■□□□□□□■■■■■□□□□□□□□□□□□■■■■■■■■■	703601740003771	CAS1 DELHI	3 (1.3)	0	0	3 (0.8)
2419^*^	■■■□□□□■■□□□□□□□□□□□□□□□□□□□□□□□□□□□□■■■■■■	703000000000371	CAS	1 (0.4)	0	0	1 (0.3)
ORPHAN 1^*^	■■■□□□□□□□□□□□□□□□□□□□□□□□□□□□□□□□□□■■■■■■■	700000000000771	CAS	2 (0.9)	0	0	2 (0.6)
ORPHAN 2^*^	■■■□□□□■■■■■■■■■■■■■■■□□□□□□□□□□□□■■■■■■■□■	703777740003761	CAS1 DELHI	1 (0.4)	0	0	1 (0.3)
ORPHAN 3^*^	■■■□□□□■■■■■■■■□□■■□□□□□□□□□□□□□□□□□□□□□□□□	703777747760371	T1	1 (0.4)	0	0	1 (0.3)
ORPHAN 4	■□□■■■■■■■■■■■■■■■■■■■■■■■■■□□□■□□□□■■■■■■■	477777777420771	H4	1 (0.4)	0	0	1 (0.3)
ORPHAN 5	■■■■■■□■■■■■■■■■■■■■■■■■■■■■■■■■□□■■■■■■■■■	773777777763771	MANU	0	2 (1.8)	0	2 (0.6)
ORPHAN 6^*^	□■■■■■■■■■■■■■■■■■■■■■■■■■■■■■■■□□□□■■□□□□■	377777777760601	T1	2 (0.9)	0	0	2 (0.6)
UNIQUE	■■■□□□□□■■■■■■■■■■■■■■□□□□□□□□□□□□■■■■■■■■■	701777740003771	UNIQUE	2 (0.9)	0	0	2 (0.6)
UNIQUE	■■□□□□□□□□□□□□□□□□□■■■■■■■■■■■■□□□□□□■□■■■■	600000377740271	UNIQUE	0	1 (0.9)	1 (1.8)	2 (0.6)
UNIQUE	■■■■■□□□□■■■■■■■■■■■■■■■■■■■■■■■□□□□■■■■■■■	760777777760771	UNIQUE	1 (0.4)	0	0	1 (0.3)
UNIQUE	■■■■■■■■■■■■■■■■■■■■□□□□□□■■□□□□■□■■□■■■■■■	777776707413771	UNIQUE	1 (0.4)	0	0	1 (0.3)
UNIQUE	■■■□□□□□■■■■■■■■■■■■■■□□□□□□□□□□□□■■■■■■■■■	703317740003771	UNIQUE	2 (0.9)	1 (0.9)	0	3 (0.8)
UNIQUE	■□□■■■■■■■■■□■■■■■■■■■■■■■■■□□□□■□■■□□□■■■■	477737777413071	UNIQUE	1 (0.4)	0	0	1 (0.3)
UNIQUE	■■■■■■■■■■■■□■■■■■■■■■■■■■■■■□□■□□□□□□□□□□□	777737777620000	UNIQUE	1 (0.4)	1 (0.9)	0	2 (0.6)
UNIQUE	■■■■■■■■■■■■■■■■■■□■□■■□□■■■□■■■□□□□■■■■■■■	777777263560771	UNIQUE	1 (0.4)	0	0	1 (0.3)
UNIQUE	■■■□□□□□□□■■■■■■■■■■■□□□□□□□□□□□□□□■■■■■□■■	700377700001751	UNIQUE	2 (0.9)	0	0	2 (0.6)
UNIQUE	■■■□□□□■■■■■□■■□□■■■■■□□□□□□□□□□□□■■■■■■■■■	703731740003771	UNIQUE	1 (0.4)	0	0	1 (0.3)
UNIQUE	■■■■■■■■■■■■■■■■■■■■□□□□□□■■□□□□■□■■□■■■■■■	777777601413371	UNIQUE	1 (0.4)	0	0	1 (0.3)
UNIQUE	□□□□□□□■■■■□■■■■■■■■■■□□□□□□□□□□□□□□■■■■■■■	003677740000771	UNIQUE	1 (0.4)	0	0	1 (0.3)
UNIQUE	■■■■■■■■■■■■■■■■■■■■■□□□■□□□□□□■□□□□■■■□■■■	777777704020731	UNIQUE	0	1 (0.9)	0	1 (0.3)
UNIQUE	■■■□□□□■■□□□□□□□□□□□□□□□□□□□□□□□□□□□■■■■■■■	703000000000771	UNIQUE	0	1 (0.9)	0	1 (0.3)
UNIQUE	■■■□■■□■■□■■■□■■■■■■■■□□□□□□□□□□□□■■■■■■■■■	733357740003771	UNIQUE	0	1 (0.9)	0	1 (0.3)
UNIQUE	■■■■■■■■□□■■■■■■□■■■■■■□■■■□□□□■□□□□■■■■■■■	776375767020771	UNIQUE	0	2 (1.8)	0	2 (0.6)
UNIQUE	■■■□□■□□■□■■■□□■■■■■■■□□■■□□■■□□□□■■■■■■■■■	711347746303771	UNIQUE	1 (0.4)	0	0	1 (0.3)
UNIQUE	■■■□■■□□□□□■■■■■■■■■■■■■■■■■■■■■□□□□■■■■■■■	730177777760771	UNIQUE	2 (0.9)	1 (0.9)	0	3 (0.8)
UNIQUE	■■■■■■□■■■□□■■■■■■■■■■□■□■■■□□□□□□■■□■■■■■■	773477753403371	UNIQUE	0	1 (0.9)	0	1 (0.3)

*SITs- that are evolved among MDR-TB isolates (second-line sensitive) from India.

**SITs-that are evolved among pre-XDR TB isolates from India.

***SITs that are evolved among XDR-TB isolates from India.

### Frequency of various Genotypes in second line drug sensitive isolates

Out of the 231 (64.3%) isolates that were found sensitive to second line drugs, 108 (46.7%) belong to the CAS lineage, 62 (26.8%) to Beijing and 18 (7.7%) to the T lineage. Seventeen (7.3%) isolates showed Unique SIT patterns, 10 (4.3%) belonged to Haarlem, 9 (3.8%) EAI, 3 (1.3%) Manu and 2 (0.8%) each belong to X and S lineages. While analysing the clustering of these strains, 207 (89.6%) strains could be grouped under 39 STs, while 7(3.4%) were identified as orphans and the rest 19 (8.2%) had unique patterns (Tables 4 and 5). Among the 39 SITs the commonest were SIT26 (CAS1_DELHI), SIT1 (Beijing) and SIT25 (CAS1_DELHI) with 65 (28.1%), 57 (24.7%) and 11 (4.7%) strains each group. Other genotypes representing fewer genotypes are given in the Table (4).

**Table 5:** Distribution and clustering pattern of MDR-TB isolates.

LINEAGE	MDR	Pre-XDR	XDR	TOTAL
BEIJING				
Clustered	3(2–57)	2(2–46)	1(8)	6(2–57)
Un-clustered	0	0	1	1
CAS				
Clustered	11(2–65)	5(2–18)	0	16(2–65)
Un-clustered	7	5	3	15
EAI				
Clustered	3(2–4)	1(2)	0	4(2–4)
Un-clustered	1	1	1	3
MANU				
Clustered	1(2)	1(2)	0	2(2)
Un-clustered	1	0	0	1
H				
Clustered	3(3–4)	1(3)	0	4(3–4)
Un-clustered	1	1	0	2
X				
Clustered	0	1(5)	0	1(5)
Un-clustered	2	1	1	4
T				
Cluster	2(2–9)	1(2)	1 (1)	4(2–9)
Un-clustered	4	5	0	9
S				
Clustered	1(2)	0	0	1(2)
Un-clustered	0	0	0	0
URAL				
Clustered	0	0	0	0
Un-clustered	0	1	1	2
ORPHAN				
Clustered	2(2)	1(2)	0	3(2)
Un-clustered	3	0	0	3
UNIQUE				
Clustered	5(2)	1(2)	0	6(2)
Un-clustered	7	6	1	14
Total				
Clustered	31(2–65)	14(2–46)	2(9)	47(2–65)
Un-clustered	26	20	8	54

### Frequency of various Genotypes in pre-XDR-MTB isolates

Out of 111 (30.9%) pre-XDR TB isolates detected by MGIT-960 DST system, the most frequent genotypes belonged to Beijing (48, 43.2%) genotype, CAS (31, 27.9%) and Unique (10, 9.1%). Other genotypes were less commonly seen and are given in Table (4). Among the 26 SITs identified, like second line drug susceptible isolates, in pre-XDR isolates also, the SIT1 (Beijing) [46; 41.4%] and SIT26 (CAS1_DELHI) [18; 16.2%] were the two most common types followed by ST119 (X1, 3.6%). But the predominance of Beijing genotype was unequivocal (26.8% v 43.2%). Of the two Orphans identified, both belonged to the CAS1_DELHI genotype. The remaining SITs represented less than 3.6% isolate (Supplementary Fig. 2).

### Frequency of various Genotypes of XDR-MTB isolates

Out of 17 (4.7%) XDR-TB isolates that were detected by the SLD MGIT-960 DST, 9 (52.9%) isolates represented as Beijing lineage, 3 (17.6%) as CAS and 1 (5.8%) each belong to EAI, X, T, Ural, and Unique patterns. Therefore, there was a clear pattern of Beijing genotype become more and more and common in isolates developing drug resistance. A total of 9 SITs were identified among 16 (94.1%) strains and one (5.8%) strain had unique spoligotype pattern. Beijing strains of SIT1 predominated [8 (47%)], whereas the remaining 15 SITS represented one (5.8%) isolate each (Table 4).

### Cluster analysis

The cluster analysis revealed that our isolates belonged to overall 47 clusters with the size of 2–65 isolates in each cluster. The highest clustering (31 clusters) was found among the MDR-TB isolates but sensitive to second-line drugs. Amongst the pre-XDR-TB isolates highest number of clusters were observed in the CAS lineage having 5 clusters (2–18 isolates in each cluster) followed by Beijing with 2 clusters of 2–46 isolates. However, in the XDR-TB isolates only one cluster was found with 8 isolates all belonging to the Beijing lineage. Isolates showing unique patterns were also found in a single cluster with two isolates in the pre-XDR TB isolates. Interestingly, most of the X, T, orphan and unique strains were un-clustered (Table 5).

**Figure 1:**
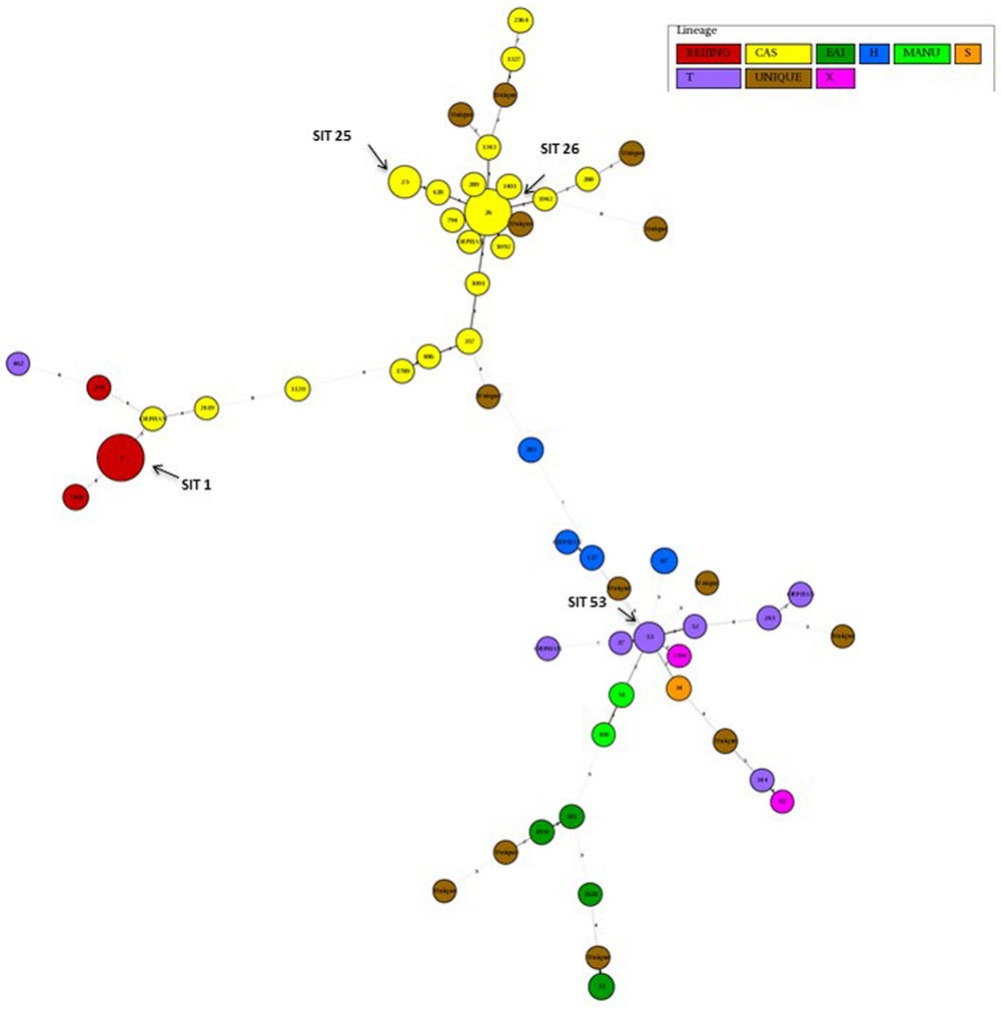
The figure shows minimum spanning tree of MDR-TB (but sensitive to second-line drug) isolates characterized by spoligotyping. Each circle represents a genotype. The distance between circles represents how closely related are different genotypes to each other.

**Figure 2:**
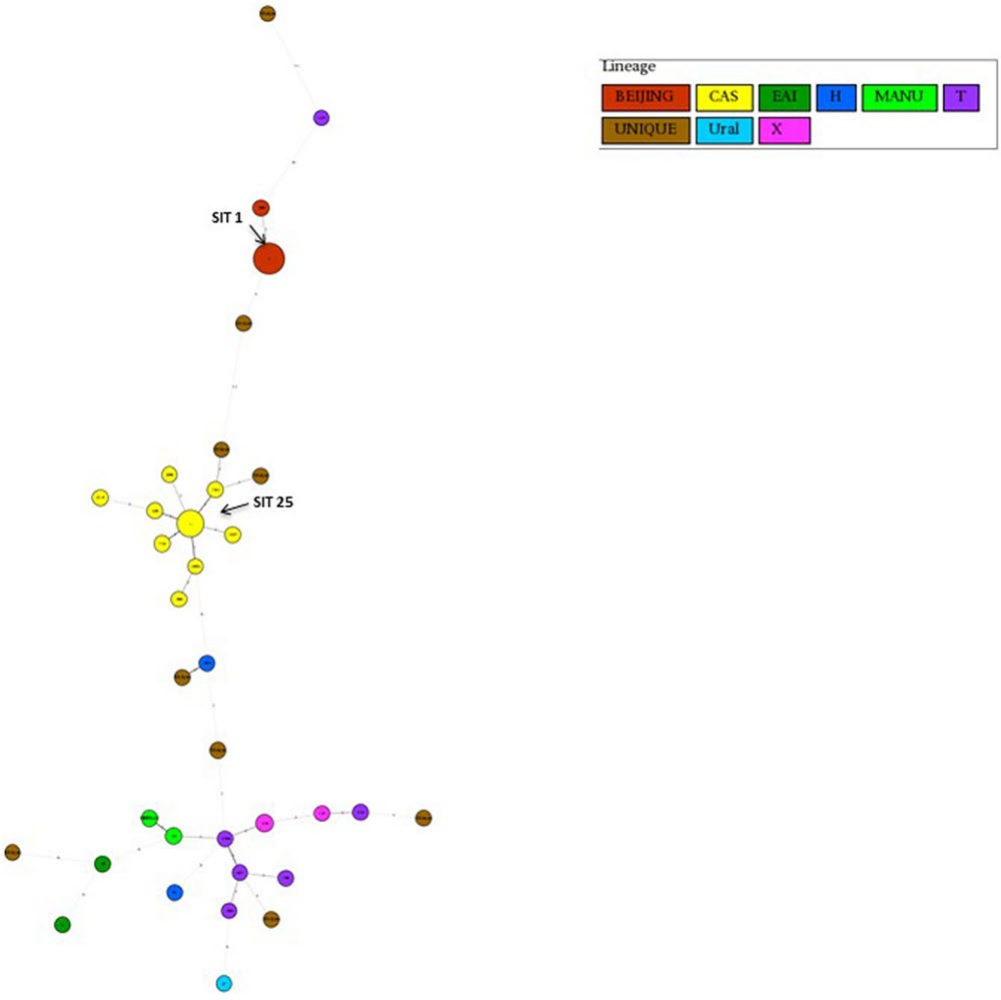
Minimum spanning tree of pre-XDR-TB isolates using the same spoligotyping method as mentioned above. Each circle represents a genotype. Each circle represents a genotype. The distance between circles represents how closely related are different genotypes to each other.

**Figure 3:**
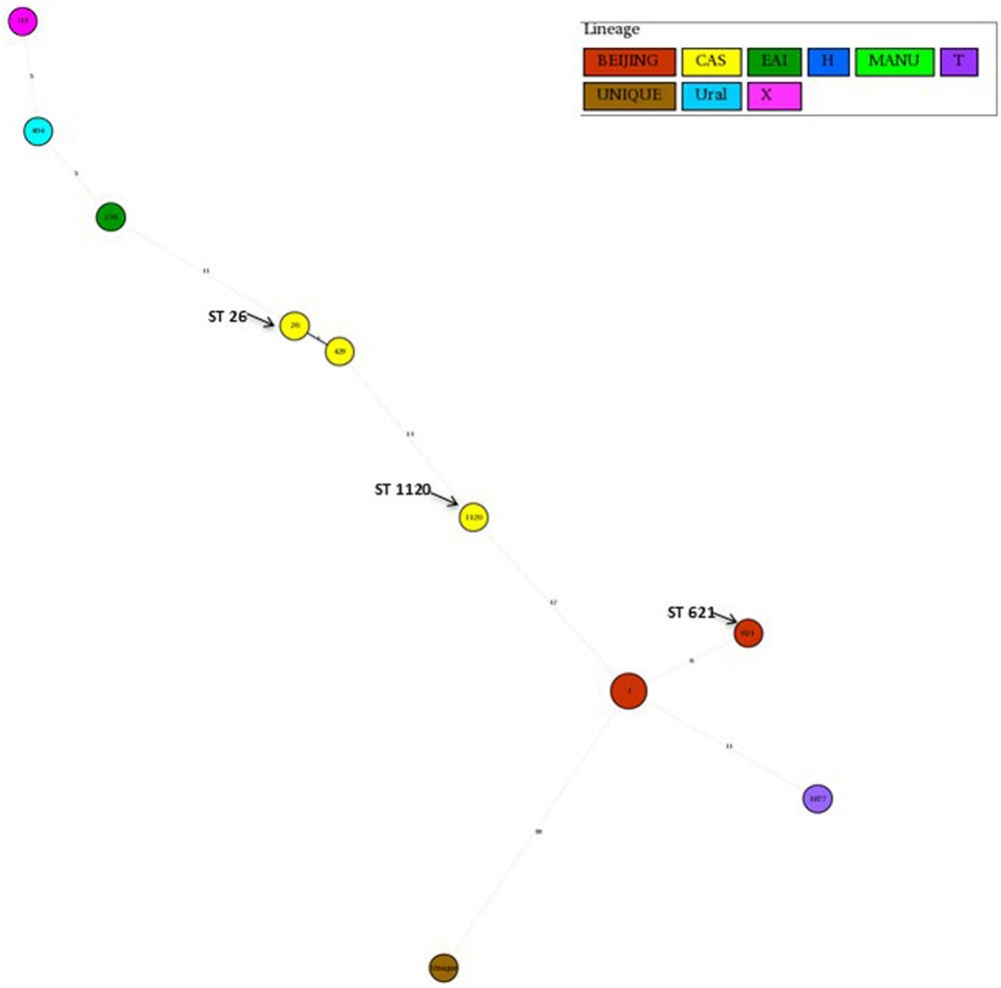
Minimum spanning tree of XDR-TB isolates using spoligotyping method as mentioned above. Each circle represents a genotype. Each circle represents a genotype. The distance between circles represents how closely related are different genotypes to each other.

Minimum Spanning Tree (MST) analysis done by using the MIRU-VNTRplus software, revealed various SITs amongst different regions showing predominant SITs and evolutionary relationship of the lineages and their SITs. MST connects each genotype based on the degree of changes required to go from one allele to another. The length of the branches denotes the distance between any two patterns whereas the intricacy of the lines indicates the number of spacers between the two patterns. The thicker lines represent 1 change while thinner ones indicate 2 or 3. The size of the circle is comparative to the total number of MTB isolates in this study. The colour of the circles represents the phylogenetic lineage to which the specific pattern belongs. Beijing patterns are circled in red and yellow indicates CAS strains. EAI strains are in dark green colour while EAI strains are in dark green colour. The Clustering and MST of MDR-TB isolates (but susceptible to second line drugs) is shown in Fig. (1). The clustering details for pre-XDR strains are given in Fig. (2). Out of 111 isolates, a total of 88 (79.3%) were grouped into 13 clusters, whereas 23 (20.7%) were non-clustered isolates of which 8 (34.8%.) were unique non-clustered isolates. In case of XDR-TB strains these details are shown in Fig. (3). Out of 17 isolates, 8 (47.1%) isolates could be grouped only in 1 cluster, whereas 9(52.9%) were non-clustered isolates of which 1 (11.1%.) was unique non-clustered isolate.

### Evolution of new share types among MDR-TB isolates

In our study 18 new SITs, were found in MDR-TB isolates, which have not been previously reported so far from India in the SITVIT2 database. Among the second line drug susceptible isolates, SIT 250 (Beijing, n = 1); SIT 462 (T1, n = 1); SIT 1628 (EAI5, n = 1); SIT 1168 (Beijing, n = 3); SIT 1394 (X1, n = 1); SIT 1401 (CAS1_DELHI, n = 3); SIT 2419 (CAS, n = 1); Orphan1 (CAS, n = 2); Orphan2 (CAS1_DELHI, n = 1); Orphan3 (T1, n = 1); Orphan 6 (T1, n = 2) were found to be evolved. New SITs, which were common in both MDR, and pre-XDR-TB isolates, included SIT 67 (H3, n = 5), SIT 243 (T1, n = 4) and SIT 794 (CAS, n = 4), respectively. Amongst the pre-XDR TB isolates, SIT 27 (Ural, n = 1); SIT 125 (T2, n = 1); SIT 190 (Beijing, n = 2); SIT 358 (T1, n = 1); SIT 798 (T1, n = 1); SIT 2147 (CAS, n = 2) and SIT 1166 (T1, n = 1) were found. In the XDR-TB isolates, SIT 464 (Ural, n = 1) was the only new SIT found to be evolved (Table 4).

### Association of genetic mutations in *gyrA* and *rrs* genes and phenotypic drug resistance

Among the FQ resistant isolates, ΔWT3-D94G (40.8%) was most prevalent mutation and the frequency of this mutation was higher in Beijing strain (45.1%) followed by CAS (27.4%), X (9.8%), Unique (7.8%), T (5.8%), Manu and H (1.9%). The ΔWT2-A90V mutation (24.8%) was most common mutation in the Beijing (32.2%) and CAS (25.8%) strains. Only Beijing strain (100%) was associated with ΔWT3-D94N/Y (4.8%) mutations. However, among the AG/CP resistant isolates, ΔWT1-A1401G (57.9%) was the most prevalent mutation and the frequency of this mutation was higher (57.9%) in Beijing strains (Table 4).

### Discussion

The first decade of this century witnessed the emergence of XDR-TB strains for which management is extremely difficult, and this imposed serious concerns for the health care systems around the world^1,5^. To develop tests to diagnose XDR-MTB strains at the earliest possible time have become an urgent need. Various research organizations are working untiringly to develop such tests and devices. A German based company developed the first version of MTBDR*sl* for rapid screening of FQ and AG/CP resistance in the MDR-TB isolates. After preliminary evaluations, in 2013, the WHO recommended use of this test to rule out the XDR-TB^27^. However, despite India being the high TB burden country, its usefulness of this test has not been evaluated on a large number of isolates. In the present work, we evaluated the efficiency of MTBDRsl V.1 in comparison to the second-line DST using MGIT-960 system, which still remain the gold standard for second line drug susceptibility testing. We also analysed for the first time, the association of various lineages of MTB with genetic mutational patterns. Even though it is a molecular test, which is prone to several procedural errors, we found that the test protocol is highly standardized giving no invalid results, which means that all (100%) tests were valid. Taking the phenotypic MGIT-960 system as the gold standard, we found very high (97%) concordance of MTBDR*sl* for detecting the second drug susceptible isolates and for FQ resistance detection (93.5%). However, more improvised version will be required to detect AG/CP resistance, where its performance was not found very high (50%). Similar findings regarding detection of AG/CP resistance have also been previously published from Spain and China reporting sensitivity of 56% and 67% respectively^28,29^. Nevertheless, the test was found extremely good (94.1%) for XDR-TB detection in our study as well as by other studies published from Serbia and Netherlands both showing 100% sensitivity^3,30^. Similar to our results the lower detection rate for EMB resistance of 56.2% was reported from China. These authors performed this test on MDR-TB isolates and emphasized identification of other mutations for detection of EMB resistance for improvement of the test^31^. Hence the identification of novel mutations outside the QRDR gene region of *gyrA* and *gyrB* and *rrs* gene is urgently needed. The new version of the assay (i.e. MTBDR*sl* V.2.0) has been recently developed by the company for improvement to overall performance of MTBDR*sl* V.1 and in particular to its sensitivity for detection of kanamycin resistance. However, at the time of study this version was not available in India^32,33^.

While correlating the association of *gyrA* and *rrs* gene mutations in various lineages^34^ we found that even though most of our isolates were from the Northern-Western part of India (270; 75.2%) where prevalence of CAS strain is predominant yet the association of Beijing isolates was statistically significantly high (*p-0*.*0006*) with *gyrA* gene mutations but insignificant (*p-0*.*079*) with *rrs* gene mutations. Beijing strains are considerably prevalent in South East Asia and North-Eastern region of India, where its is posing a major concern due to its high prevalence among the MDR-TB patients^19,31,35,36^. However, the recent studies from India show that this strain is spreading fast to other parts of India and neighbouring countries^14^, which can be warning signal to the TB control programme managers. Within the Beijing genotype SIT1 was most predominant share type amongst the pre-XDR and XDR-TB isolates in our study. SIT1 was also reported predominant among XDR- TB isolates from Africa (34%) and Russia (9.5%)^37,38^. We for the first time reported 18 new SITs and 5 Orphans in the Indian MDR-TB isolates, though these types have previously been reported from other countries, like SIT67 (1.4%) from United States and Mexico, SIT243 (1.1%) from Zambia, Vietnam and Italy, SIT 794 (1.1%) from Bangladesh, Pakistan and United States^39–43^. The two newly evolving SITs in the Beijing genotype [SIT 1168 (0.8%) and SIT 190 (0.5%)], were found only in the MDR and pre-XDR TB isolates. These SITs have been reported from Unites States, Thailand, Japan, Vietnam and China, but never from India^41,43^. The Ural SIT 464 was the only strain evolved among the XDR-TB isolate which have never been reported from India but only from United States of America^39^. Evolution of new share types among the MDR, pre-XDR and XDR-TB isolates from India indicates these strains got transmitted to India through migration of population from such geographical regions in recent years^14^. We also report association of Beijing genotypes with very high frequency of 2 *gyrA* gene mutations-ΔWT3-D94G and ΔWT2-A90V (Table 4).

In conclusion, even though our study had some limitations such as not being able to monitor the progression of disease and treatment outcome of patients due to the use of archival MDR culture isolates, our study signifies that MTBDR*sl* V1 is a good diagnostic tool for the detection of pre-XDR and XDR-TB. We propose that MTBDR*sl* V1 should be used on all MDR-TB isolates in place of phenotypic culture DST methods, till its second version is made available, in the programmatic mode. This strategy is more pertinent for countries and regions where pre-XDR and XDR-TB prevalence is high. We also conclude that more prospective genotyping studies along with next-generation sequencing methods be implemented in order to ensure the understanding of the vulnerability of some genotypes to the drug resistance development and the molecular mechanisms leading to the emergence of pre-XDR and XDR-TB strains.

## Electronic supplementary material

Supplementary Information
