# Supplementary material for: Association of gyrA and rrs gene mutations detected by MTBDRsl V1 on Mycobacterium tuberculosis strains of diverse genetic background from India
Source: Sci Rep. 2018 Jun 18;8:9295. doi: 10.1038/s41598-018-27299-z (PMC6006251; doi:10.1038/s41598-018-27299-z)
Supplement: Supplementary file 1 — Supplementary Information [file 41598_2018_27299_MOESM1_ESM.pdf]

Association of *gyrA* and *rrs* gene mutations detected by MTBDR*sl*V1 on *Mycobacterium tuberculosis* strains of diverse genetic background from India

Syed Beenish Rufai, Jitendra Singh, Parveen Kumar, Purva Mathur, Sarman Singh#

[1]Division of Clinical Microbiology and Molecular Medicine, Department of Laboratory Medicine, All India Institute of Medical Sciences New Delhi, India.

Running title: Genotype MTBDR*sl* on MDR-TB isolates

Key words: Line Probe Assay, Spoligotyping, Genotyping, Beijing, Second Line DST

# Corresponding Author:

Prof. Sarman Singh, MD

Phone: 91-11-2658-8484/26594977

Fax: 91-11-2658-8663/ 2658-8641

Email: sarman_singh@yahoo.com, sarman.singh@gmail.com

Supplementary table 1. Association of first line drug resistant patterns with second line drug susceptibility test by Bactec MGIT-960.

SIRE drug susceptibility patterns by MGIT 960						Second line dug susceptibility patterns obtained by MGIT 960					
**S**	**I**	**R**	**E**	**n**	**%**	**OFX**	**AMK**	**KAN**	**CAP**	**n**	**%**
S	R	R	S	115	32	R	S	S	S	21	18.3
						R	R	R	S	2	1.7
						R	R	R	R	1	0.9
						S	S	S	S	91	79.1
S	R	R	R	35	9.7	R	S	S	S	8	22.8
						R	R	R	R	2	5.7
						S	S	S	S	25	71.4
S	S	R	S	2	0.5	S	S	S	S	2	100
R	R	R	S	99	27.5	R	S	S	S	27	27.3
						R	R	R	S	2	2
						R	R	R	R	2	2
						S	S	S	S	68	68.7
R	R	R	R	107	29.8	R	S	S	S	54	50.5
						S	R	R	S	1	0.9
						R	S	R	S	1	0.9
						R	R	R	S	2	1.9
						R	R	R	R	4	3.7
						S	S	S	S	45	42.1
S	S	R	R	1	0.3	R	R	R	R	1	100
Total				359		OFX^{Res} 117	AMK^{Res}17	KAN^{Res}18	CAP^{Res} 10		

OFX^{Res} – Ofloxacin resistant; AMK^{Res}– Amikacin resistant; KAN^{Res}– Kanamycin resistant; CAP^{Res} Capreomycin resistant

Supplementary Figure-1.

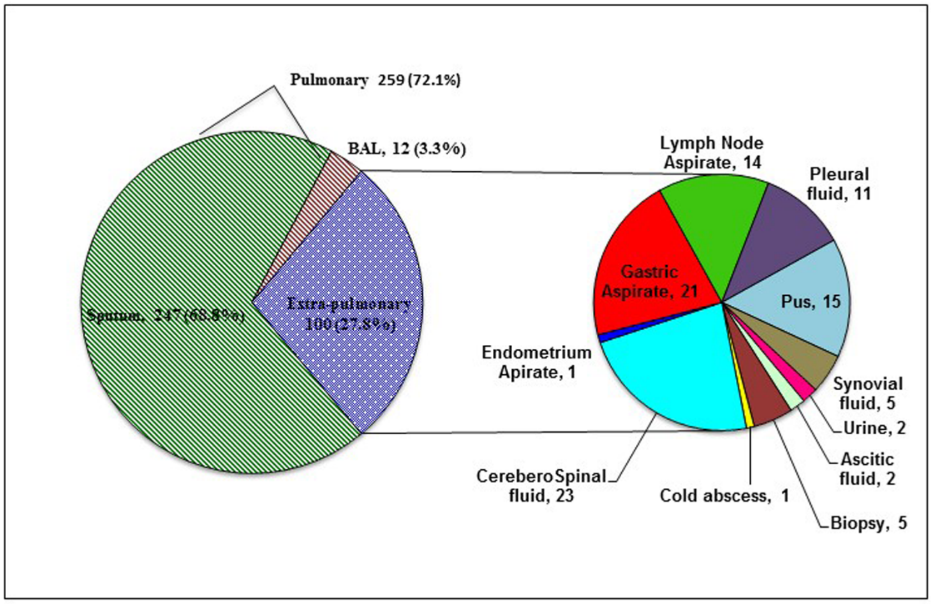

Showing the distribution of Pulmonary (259; 72.1%) and Extra-pulmonary (100; 27.8%) isolates of MDR-TB used in the study. Of total 259 (72.1%) pulmonary TB isolates, 247; 68.8% were sputum and 12 (3.3%) were BAL. Among 100 (27.8%) Extra-pulmonary TB isolates, majority of isolates were from CSF (23%), GA (21%); LNA (14%) and PF (11%) and remaining isolates were from biopsy and other body fluids.

Supplementary Figure 2.

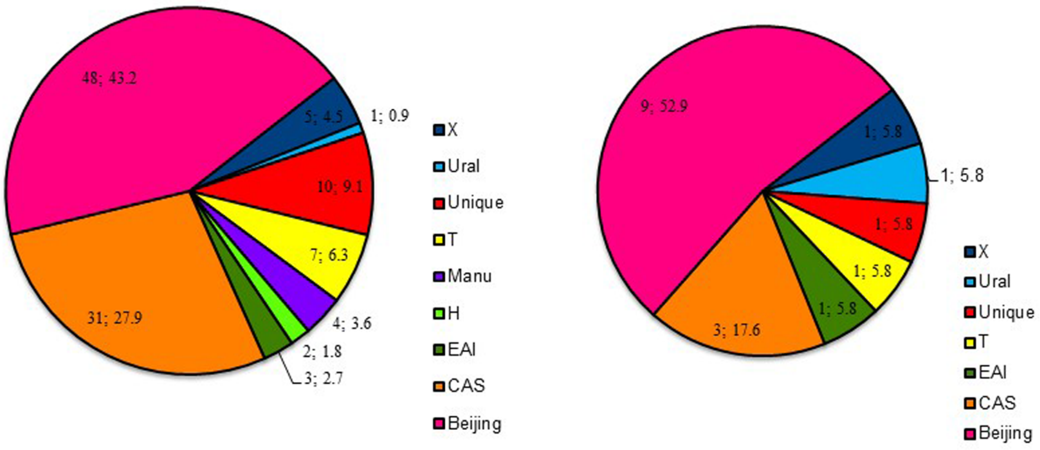

Showing distribution of different genotypes among 111 pre-XDR-TB isolates (a); and distribution of different genotypes among 17 XDR TB isolates (b).
